# Navigated robot-guided pedicle screws placed successfully in single-position lateral lumbar interbody fusion

**DOI:** 10.1007/s11701-019-01034-w

**Published:** 2019-10-17

**Authors:** Kade T. Huntsman, Jessica R. Riggleman, Leigh A. Ahrendtsen, Charles G. Ledonio

**Affiliations:** 1 Salt Lake Orthopaedic Clinic, Suite 500, 1160 East 3900 South, Salt Lake City, UT 84124 USA; 2 Musculoskeletal Education and Research Center (MERC), A Division of Globus Medical, Inc., 2560 General Armistead Avenue, Audubon, PA 19403 USA

**Keywords:** Robot, Navigation, Pedicle screw accuracy, Robot assistance, Minimally invasive, Spine surgery

## Abstract

Minimally invasive lateral interbody fusion has distinct advantages over traditional posterior approaches. When posterior stabilization is needed, percutaneous placement of pedicle screws from the lateral decubitus position may potentially increase safety and improve operative efficiency by precluding the need for repositioning. However, safe placement of pedicle screws in the lateral position remains technically challenging. This study describes the pedicle screw placement of single-position lateral lumbar interbody fusion (SP-LLIF) cases in which navigated robotic assistance was used. A single-surgeon, single-site, retrospective Institutional Review Board-exempt review of the first 55 SP-LLIF navigated robot-assisted spine surgery cases performed by the lead author was conducted. An orthopaedic surgeon evaluated screw placement using plain film radiographs. In addition, pedicle screw malposition, reposition, and return to operating room (OR) rates were collected. In the first 55 SP-LLIF cases, 342 pedicle screws were placed. The average patient age and body mass index were 67 years and 29.5 kg/m^2^, respectively. Of the 342 screws placed, 4% (14/342) were placed manually without the robot, due to surgeon discretion. Of the 328 screws placed with the robot, 2% (7/328) were repositioned based on the surgeon’s discretion, resulting in a 98% navigated robot-assisted pedicle screw placement success rate. In this cohort there were no revisions due to malpositioned screws. No complications due to screw placement were reported. This study demonstrates a high level (98%) of successful surgeon-assessed pedicle screw placement in minimally invasive navigated robot-assisted SP-LLIF, with no malpositions requiring a return to the OR.

## Introduction

Open lumbar interbody fusion is a popular method for treating patients with spinal back and leg pain who have failed conservative management. However, this technique is associated with decreased functional outcomes due to greater muscle and tissue disruptions, which may lead to a higher complication rate [1–3]. Minimally invasive spine surgery has become more widespread due to perceived advantages such as minimal muscle disruption, decreased blood loss, shorter operating room (OR) time, shorter hospital stays, and lower complication rates [3–7].

Minimally invasive lateral lumbar interbody fusion is a retroperitoneal approach that allows the placement of a larger implant, allowing for a larger fusion bed. When posterior stabilization is needed, percutaneous placement of pedicle screws from the lateral decubitus position precludes the need for repositioning, potentially increasing safety and reducing OR time, cost, and radiation. However, achieving safe placement of pedicle screws in the lateral position remains technically challenging.

There is of high interest in the emerging field of robot-assisted spine surgery with navigation; however, studies evaluating the safety and accuracy of this technique are needed. This study describes the pedicle screw placement in single-position lateral lumbar interbody fusion (SP-LLIF) cases with navigated robotic assistance.

## Methods

This is a single-surgeon, single-site, retrospective Institutional Review Board-exempt review of the first 55 consecutive SP-LLIF navigated robot-assisted spine surgery cases. The demographic, intraoperative, and perioperative data of 55 patients who underwent lumbosacral pedicle screw placement with a minimally invasive navigated robot-assisted pedicle screw positioning system (ExcelsiusGPS^®^, Globus Medical, Inc. Audubon, PA, USA) were reviewed. An orthopaedic surgeon evaluated screw placement using plain film radiographs. Data on pedicle screw malposition, reposition, and return to OR rates were collected.

### Preoperative CT workflow

A computed tomography (CT) scan of the spinal levels in the operative field was taken prior to the patient entering the OR, and screw placement planning was completed. The CT data set was transferred into the robotic positioning system, and then registration was completed for vertebral levels.

### Intraoperative CT workflow

In intraoperative CT mode, the image coordinate system was obtained from a portable intraoperative CT (e.g. O-arm, Medtronic SNT, Louisville, CO, USA) or standard CT scan was taken at the time of surgery, with the patient already in position on the OR table. Spinal levels were identified and a CT scan was taken. Pedicle screw trajectories were planned and saved.

### Surgical technique

**Fig. 1:**
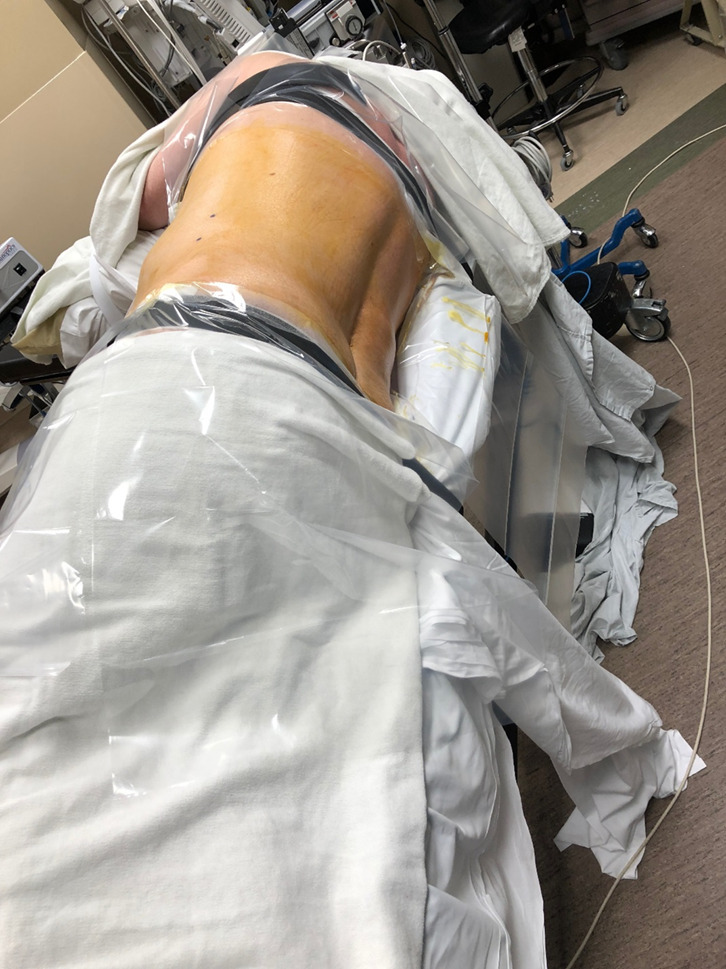
Patient positioning for SP-LLIF

**Fig. 2:**
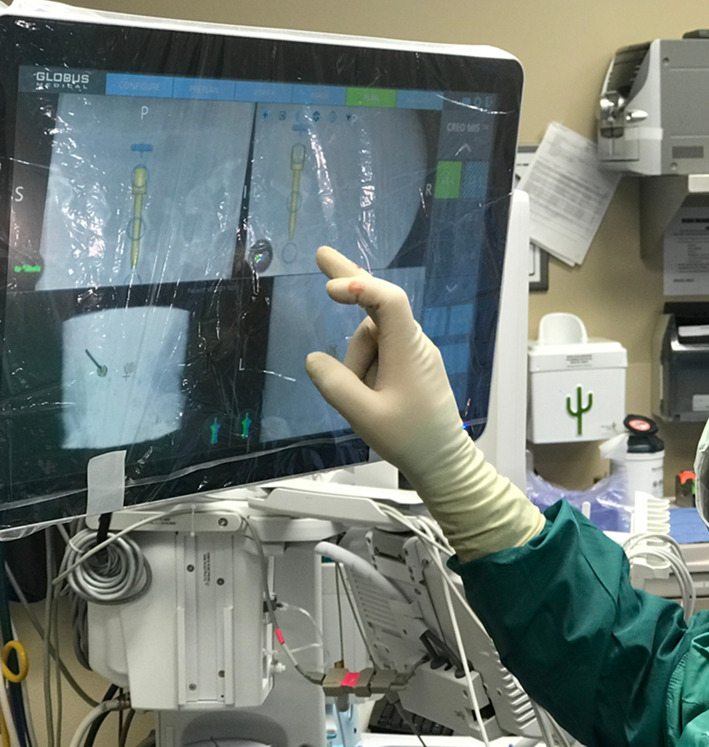
Intraoperative pedicle screw placement planning

**Fig. 3:**
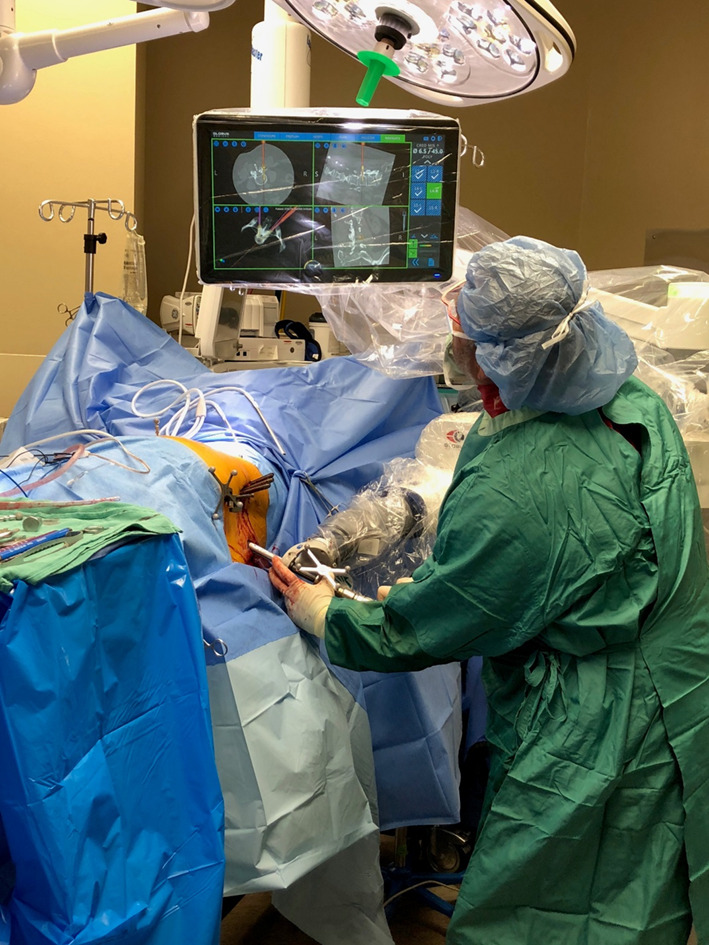
Minimally invasive lumbosacral pedicle screw placement with a navigated robot-assisted positioning system

The patient was positioned in the lateral decubitus position (Fig. 1). A dynamic reference base and a surveillance marker were placed. An intraoperative CT was taken and registered with the software. Pedicle screw trajectory planning was performed (Fig. 2). A surgeon-controlled foot pedal activated and positioned the robot arm to the planned pedicle trajectory. Stab incisions were made on the skin using a scalpel. Pedicle screws were inserted under neuromonitoring using navigated instruments guided by the robotic arm (Fig. 3). This sequence was repeated until all pedicle screws had been placed.

Following pedicle screw placement, lumbar interbody fusion was performed using the lateral approach. The endplates were prepared and an interbody spacer of appropriate size was manually inserted. Rods were then placed in a standard fashion, with the patient remaining in the lateral position. Locking caps were set once the rods were in their proper position.

**Fig. 4:**
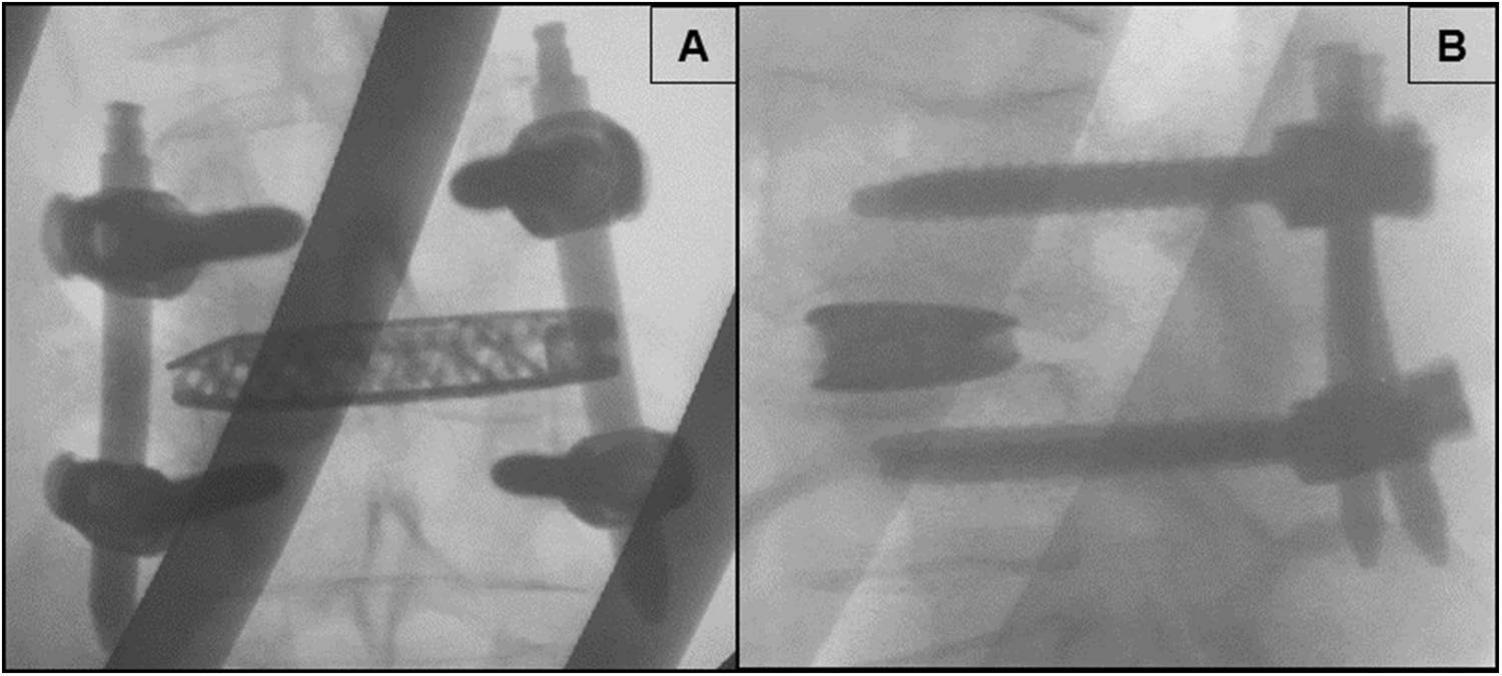
Postoperative anteroposterior (**a**) and lateral (**b**) fluoroscopic images of a one-level MIS SP-LLIF

Intraoperative fluoroscopy images were taken to verify the pedicle screw, interbody spacer, and rod position. Pedicle screw placement was qualitatively assessed using postoperative X-rays (Fig. 4).

### Statistical analysis

The statistical analysis was performed using IBM^®^ SPSS^®^ Version 25 software (IBM^®^ Corp.; Armonk, NY, USA). Data were presented as mean ± standard deviation. Statistical significance is shown at* P* < 0.05.

## Results

### Baseline characteristics

The average patient age was 67 years and 49% (27/55) were female. The average BMI was 29.5 kg/m^2^. Fifteen patients (27%) were either former or current smokers. The majority of patients were either retired (53%) or worked full time (31%) (Table 1).

**Table 1:** Baseline characteristics

Parameter	Overall
Number of patients	55
Gender	
Female, *n* (%)	27 (49.1)
Male, *n* (%)	28 (50.9)
Age, mean (SD, range)	66.8 (9.1) (43–82)
BMI, mean (SD, range)	29.5 (6.2, 17–44)
Smoker, *n* (%)	
Never	40 (72.7)
Former	10 (18.2)
Current	5 (9.1)
Work status, *n* (%)	
Retired	29 (52.7)
Full time	17 (30.9)
Part time	3 (5.5)
Unemployed	1 (1.8)
Disabled	3 (5.5)
Unknown	2 (3.6)

### Surgical data

Of the 55 patients, 171 levels were treated, and 30% (52/171) were treated at L4. Thirty-five percent (19/55) of patients underwent a two-level SP-LLIF. The average estimated blood loss was 117 cc. The average overall operative time was 155 min. The average length of hospital stay was 2.9 days. The most common workflow with the minimally invasive navigated robot-assisted pedicle screw positioning system was intraoperative CT (75%) (Table 2).

**Table 2:** Surgical data

Parameter	Overall
Levels treated, *n* (%)	
L1	6 (3.5)
L2	22 (12.9)
L3	43 (25.1)
L4	52 (30.4)
L5	42 (24.6)
S1	6 (3.5)
Number of levels treated	
2	19 (34.5)
3	16 (29.1)
4	16 (29.1)
5	3 (5.5)
6	1 (1.8)
Mean estimated blood loss, *n* (SD) (cc)	117.4
Mean operative time, *n* (SD) (min)	155.7 (42.0)
Mean length of hospital stay, *n* (SD) (days)	2.9 (1.3)
Workflow, *n* (%)	
Intraoperative CT	41 (74.5)
Preoperative CT	14 (25.5)

### Pedicle screw positioning

In the first 55 SP-LLIF cases, 342 pedicle screws were placed. Of the 342 screws placed, 4% (14/342) were placed manually, without the robot due to surgeon discretion. Of the 328 screws placed with the robot, 2% (7/328) were repositioned based on the surgeon’s discretion, resulting in a 98% screw placement success rate for navigated robot-assisted pedicle screw placement. In this cohort there were no revisions due to malpositioned screws. No complications due to screw placement were reported.

## Discussion

This study demonstrates a high screw placement success rate in SP-LLIF with navigated robot-assisted spine surgery in the lumbosacral spine. High revision rates or neurological injuries with conventional freehand and two-dimensional fluoroscopy-guided pedicle screw placement has led to the development of multiple approaches to reduce complications and improve the accuracy of pedicle screw placement [8, 9]. The most recently introduced method is minimally invasive robot-assisted navigated pedicle screw placement using the SP-LLIF approach.

The lateral approach facilitates thorough intervertebral disc preparation, creates a large surface area for fusion, and allows placement of a large elongated interbody implant while maintaining the annulus and the anterior longitudinal ligament [10–12]. Minimal literature is available on the success rate of pedicle screw placement using a SP-LLIF approach, so comparison to published literature is challenging.

The advantages of minimally invasive surgery include less disruption of the paraspinal muscles and sparing of the posterior spinal column, as well as reduction of postoperative pain, operative time, and length of hospital stay. Wide exposure of the disc space in any anterolateral approach allows for the insertion of large interbody spacers, which is not possible with posterior or transforaminal lumbar interbody fusion due to proximity to the thecal sac and neural elements. An LLIF allows for the insertion of a long and wide interbody spacer that reaches the endplate diaphysis on both sides, allowing for a larger surface area for indirect decompression [10, 12, 13]. Literature has shown that larger anterior-posterior spacer dimensions resulted in a significantly improved and sustained restoration of disc and neuroforaminal height [13, 14].

Literature on pedicle screw placement success rate is variable. Santos et al. [15] studied the intraoperative revision and return to surgery rates for 988 navigated lumbar pedicle screws compared to navigated open and percutaneous techniques. They demonstrated an intraoperative revision rate of navigated lumbar pedicle screws of 4.6%.

The SP-LLIF approach has not gained widespread approval. Reports of neurological complications to the lumbar plexus range from 0.7-30% in the literature, which suggests inconsistent reporting [12]. To reduce operative time and radiation exposure, various forms of supplemental fixation have been proposed so the patient will not require repositioning, such as integrated plate fixation, unilateral pedicle screw fixation, and spinous process plate fixation [10]. Ziino et al. [16] showed that single-position lateral pedicle screw fixation following lateral interbody fusion decreases operative time compared to dual-position surgery without compromising complication rates and radiographic or perioperative outcomes. In a retrospective review, Blizzard et al. [10] investigated the short-term outcomes and perioperative complications from a series of patients who underwent LLIF with bilateral percutaneous pedicle screw placement in the lateral decubitus position. A 5.1% overall breach rate and a 2.8% reoperation rate were reported. Generally, revision surgery rates are difficult to track, hence the lack of published data.

Inserting screws and interbody spacers using the SP-LLIF approach can be challenging for inexperienced surgeons due to the patient’s position. The robot used in this study is well-suited for the SP-LLIF approach, providing rigidity and stability to maintain the optimal trajectory during pedicle screw placement [17].

### Study limitations

Although this is a single-surgeon, single-site, retrospective study without a comparative control, the results are consistent with findings from the literature. This study forms the foundation for future studies with a higher level of evidence. Comparative studies with larger sample sizes and longer follow-up are needed to determine the effectiveness of SP-LLIF versus repositioning after LLIF.
